# Varying Oxygen Partial Pressure Elicits Blood-Borne Microparticles Expressing Different Cell-Specific Proteins—Toward a Targeted Use of Oxygen?

**DOI:** 10.3390/ijms23147888

**Published:** 2022-07-17

**Authors:** Costantino Balestra, Awadhesh K. Arya, Clément Leveque, Fabio Virgili, Peter Germonpré, Kate Lambrechts, Pierre Lafère, Stephen R. Thom

**Affiliations:** 1 Environmental, Occupational, Aging (Integrative) Physiology Laboratory, Haute Ecole Bruxelles-Brabant (HE2B), 1180 Brussels, Belgium; clement.leveque.kinepro@gmail.com (C.L.); pgermonpre@gmail.com (P.G.); klambrechts@he2b.be (K.L.); plafere@he2b.be (P.L.); 2 Anatomical Research and Clinical Studies, Vrije Universiteit Brussels (VUB), 1090 Brussels, Belgium; 3 DAN Europe Research Division (Roseto-Brussels), 1160 Brussels, Belgium; 4 Motor Sciences Department, Physical Activity Teaching Unit, Université Libre de Bruxelles (ULB), 1050 Brussels, Belgium; 5 Department of Emergency Medicine, University of Maryland School of Medicine, Baltimore, MD 21201, USA; aarya@som.umaryland.edu (A.K.A.); sthom@som.umaryland.edu (S.R.T.); 6 Council for Agricultural Research and Economic-Food and Nutrition Research Centre (C.R.E.A.-AN), 00187 Rome, Italy; fabio.virgili@crea.gov.it; 7 Hyperbaric Centre, Queen Astrid Military Hospital, 1120 Brussels, Belgium

**Keywords:** hypoxia, hyperoxia, hyperbaric oxygen, cellular reactions, decompression sickness, diving, altitude, normobaric oxygen paradox, hyperoxic-hypoxic paradox

## Abstract

Oxygen is a powerful trigger for cellular reactions, but there are few comparative investigations assessing the effects over a large range of partial pressures. We investigated a metabolic response to single exposures to either normobaric (10%, 15%, 30%, 100%) or hyperbaric (1.4 ATA, 2.5 ATA) oxygen. Forty-eight healthy subjects (32 males/16 females; age: 43.7 ± 13.4 years, height: 172.7 ± 10.07 cm; weight 68.4 ± 15.7 kg) were randomly assigned, and blood samples were taken before and 2 h after each exposure. Microparticles (MPs) expressing proteins specific to different cells were analyzed, including platelets (CD41), neutrophils (CD66b), endothelial cells (CD146), and microglia (TMEM). Phalloidin binding and thrombospondin-1 (TSP), which are related to neutrophil and platelet activation, respectively, were also analyzed. The responses were found to be different and sometimes opposite. Significant elevations were identified for MPs expressing CD41, CD66b, TMEM, and phalloidin binding in all conditions but for 1.4 ATA, which elicited significant decreases. Few changes were found for CD146 and TSP. Regarding OPB, further investigation is needed to fully understand the future applications of such findings.

## 1. Introduction

Oxygen (O_2_), which belongs to the WHO list of essential medicines, has long been recognized as a common treatment for both acute and chronic diseases, and is widely applied from pre-hospital emergency medical services to home oxygen therapy [1]. Its main therapeutic objective is to correct tissue or cellular hypoxia [2]. However, pure O_2_ breathing is not only devoted to patients needing oxygen support. Indeed, other therapeutic uses of oxygen need to be considered. In those therapies, oxygen is considered as a drug capable of inducing a targeted clinical response, such as Hyperbaric Oxygen Therapy (HBOT) [3,4] or in therapies using the “Normobaric Oxygen Paradox” or the “Hyperoxic-Hypoxic Paradox” [5,6,7,8]. Moreover, O_2_ breathing goes beyond mere therapeutic use. Breathing an O_2_ mixture at different concentrations, either hypoxic or hyperoxic, has been used for sport training [9], cardiovascular conditioning [10,11], or before extreme environmental exposure, such as SCUBA diving [12,13], military high-altitude free fall [14,15], or space flight [16,17], to avoid the occurrence of decompression sickness (DCS).

DCS arises when tissues become supersaturated with metabolically inert gases. On decompression, Nitrogen (N_2_) or similar gases diffuse from sites of high concentration as a function of both the pressure gradient and blood flow, which can induce vascular gas emboli (VGE), a key element in the development of DCS. Indeed, the amount of VGE is statistically related to the risk of DCS [18]. Conversely, the absence of detectable VGE is correlated with a very low probability of DCS in both hyperbaric [19] or hypobaric [20] settings, hence the development of pre-conditioning strategies that aim to reduce VGE production. Oxygen pre-breathing (OPB), a standard approach to remove dissolved N_2_ from tissues in anticipation of exposures to sub-normal pressures associated with high-altitude aviation and extra-vehicular transits while in space, is one of those strategies [21]. OPB has been associated with a decreased incidence of DCS, especially when combined with moderate exercise [22]. However, although DCS risk is lowered, OPB does not seem to alter the time when VGE is first detected in decompressed research subjects, except in small animal research where the metabolic rate is different [18,23,24]. This is interpreted as indicating that the number of bubble nucleation sites, so-called micronuclei, present at baseline is not clearly influenced by varying the O_2_ concentration, but N_2_ mobilization or ‘wash-out’ decreases bubble formation except for a limited population of such nuclei [18]. There are also alternative strategies that appear to diminish bubble micronuclei [25]. These issues highlight the complexity of the physio-pathological mechanisms related to DCS.

The literature has identified several contributing factors to pressure exposures and DCS, such as vascular dysfunction, oxidative stress, and blood-borne microparticles (MPs) [26,27], which have been considered potential targets for pre-conditioning interventions. MPs are of particular interest since a growing body of data suggest that they are a potential bubble nucleation site and play a role in DCS pathophysiology [27,28,29,30,31]. MPs are 0.1–1 µm vesicles generated by an outward budding of the plasma membrane in a process that results in the surface expression of phosphatidylserine. As with most types of extracellular vesicles, MPs are found in all body fluids and increase in association with most human disease and injuries [32]. They are generated by virtually all cells, can be beneficial or exacerbate pathology, and exert effects due to the content of nucleic acids, inflammatory mediators, and enzymes or organelles that generate free radicals [33,34,35,36].

Oxidative stress is also considered among the issues related to DCS, especially with OPB in mind. It is known to occur with diving and documented as the upregulation of antioxidant genes and elevation of plasma and intracellular antioxidant enzyme levels [37,38,39,40,41,42]. High-pressure exposures also increase the number of MPs in human divers, marine mammals, and small animals used in models of DCS [27,28,29,30,43,44,45,46,47]. Studies with isolated human and murine neutrophils demonstrate that MP production is an oxidative stress response [48].

Since OPB protocols were developed based on pragmatic factors and the limitations of resources—such as those present during space flight—with only a view toward N_2_ removal, the aim of this investigation was to evaluate the impact of varying concentrations of O_2_ on the number of blood-borne MPs in a group of human research subjects. We considered that examining MP responses may offer more objective criteria for choices of O_2_ partial pressure.

However, when it comes to analyzing the biological responses to oxygen level variation, the trade-off between hypoxia and hyperoxia is not obvious. Large deviations from normoxia generally lead to increased oxidative stress, while the slight modulation of oxygen levels can enhance the antioxidant defenses [1,49]. We therefore investigated extremes, from 0.1 ATA (Summit of Kilimanjaro (5791 m)) to 2.5 ATA (therapeutic hyperbaric oxygen sessions), and several intermediate oxygen levels relevant to high-altitude exposure (2400 to 2700 m) or O_2_ levels used during closed circuit rebreather (CCR) diving—either in recreational, technical, or military diving (0.15, 0.30, 1.0, and 1.4 ATA) [12,50,51].

## 2. Results

### 2.1. Microparticles Elicited after One Hour of Different Oxygen Exposures

Research subjects had blood samples obtained prior to and at two hours after a one-hour exposure to various O_2_ partial pressures. MPs were identified based on size and surface expression of annexin V (a protein that binds to phosphatidylserine at the particle surface). 

**Figure 1:**
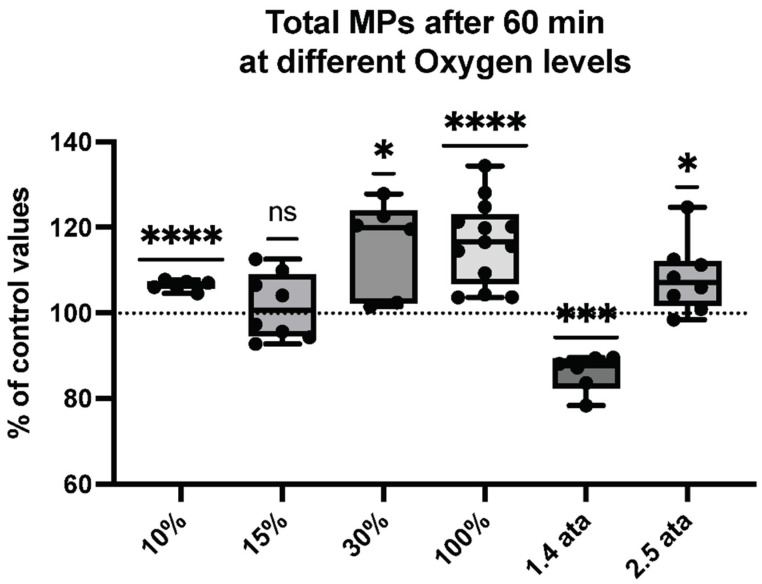
Total microparticle response following different oxygen levels. Box and Whisker plots indicating median, 1st quartile, 3rd quartile, interquartile range, min., and max. in comparison to baseline (before oxygen exposure), which was set at 100%. (One-sample *t* test: **** *p* < 0.0001, *** *p* < 0.001, * *p* < 0.05, ns = non-significant).

Figure 1 illustrates the changes in the number of blood-borne MPs. Elevations were found across the range of hypo- to hyperoxic exposures, with significant elevations following 10%, 30%, 100%, and 2.5 ATA. Breathing 15% oxygen elicited no change, while 1.4 ATA is the only O_2_ level showing a significant decrease in MP production.

### 2.2. Microparticles Expressing Proteins from Platelets, Neutrophils, Endothelial Cells, and Microglia after One Hour of Different Oxygen Exposures

The expression of antigens on the MPs surface were probed to evaluate cells generating MPs and several cells’ signaling proteins. 

**Figure 2:**
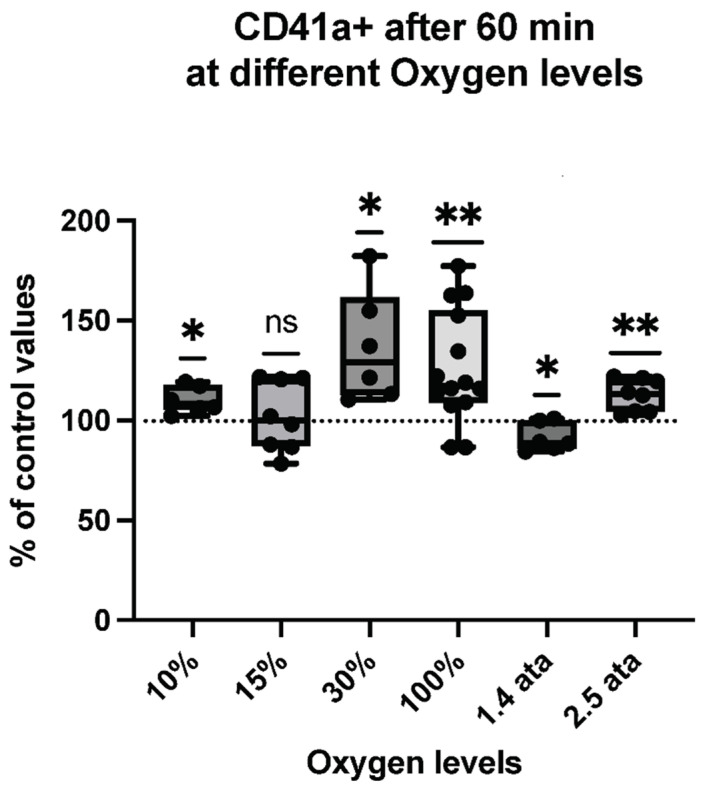
CD41+ response following different oxygen levels exposure. Box and Whisker plots indicating median, 1st quartile, 3rd quartile, interquartile range, min., and max. in comparison to baseline (before oxygen exposure), which was set at 100%. (One-sample *t* test: ** *p* < 0.01, * *p* < 0.05, ns = non-significant).

**Figure 3:**
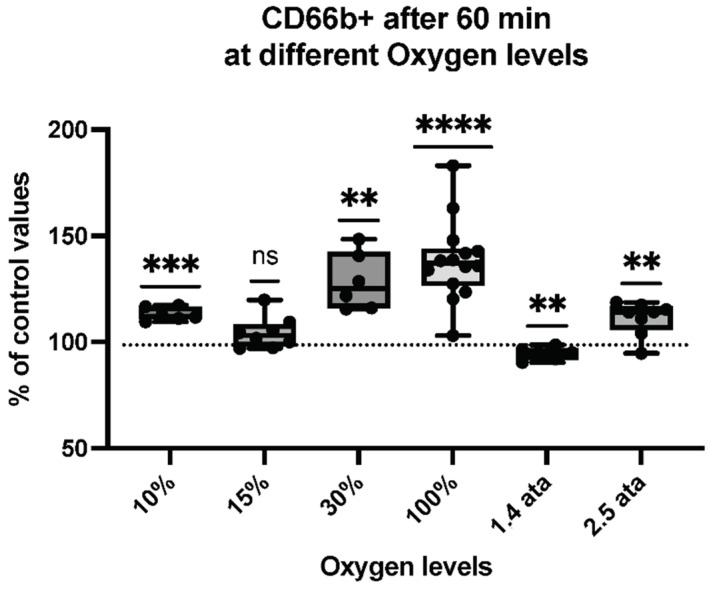
CD66b+ response following different oxygen levels exposure. Box and Whisker plots indicating median, 1st quartile, 3rd quartile, interquartile range, min., and max. in comparison to baseline (before oxygen exposure), which was set at 100%. (One-sample *t* test: **** *p* < 0.0001, *** *p* < 0.001, ** *p* < 0.01, ns = non-significant).

**Figure 4:**
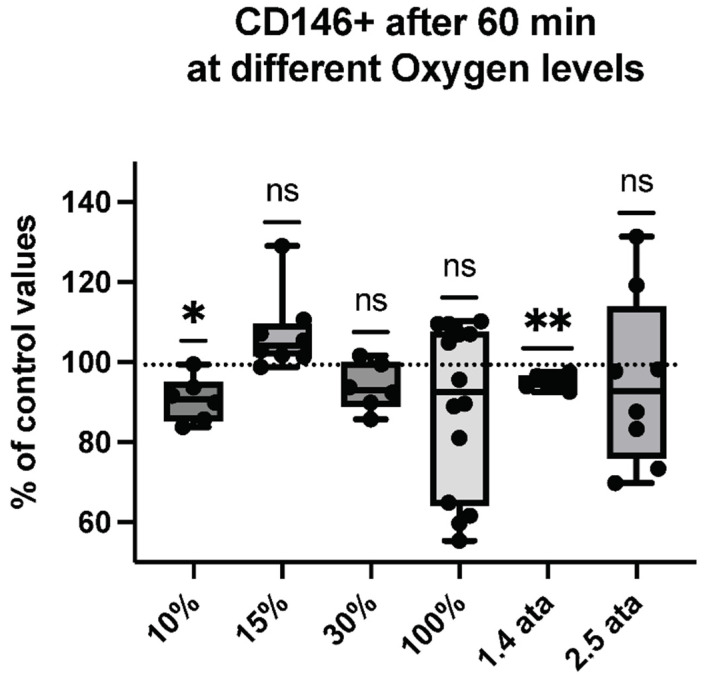
CD146+ response following different oxygen levels exposure. Box and Whisker plots indicating median, 1st quartile, 3rd quartile, interquartile range, min., and max. in comparison to baseline (before oxygen exposure), which was set at 100%. (One-sample *t* test: ** *p* < 0.01, * *p* < 0.05, ns = non-significant).

**Figure 5:**
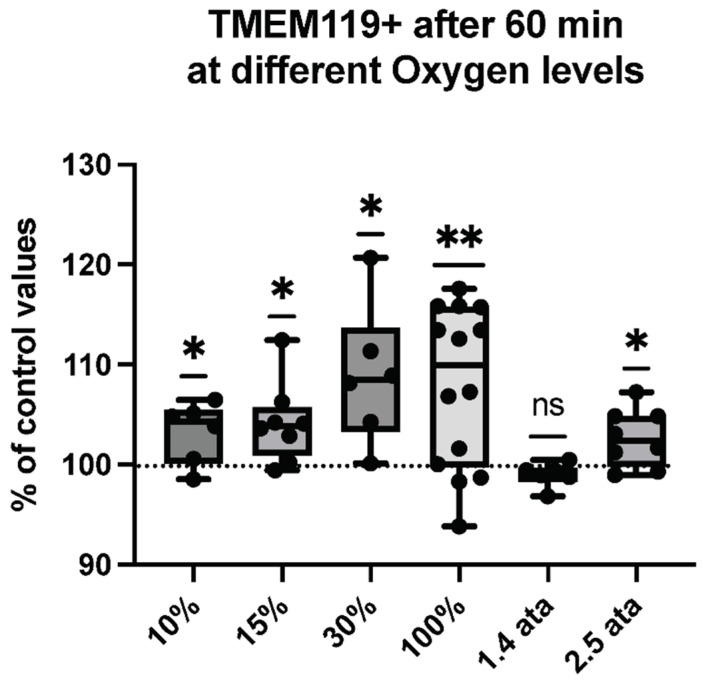
TMEM119+ response following different oxygen levels exposure. Box and Whisker plots indicating median, 1st quartile, 3rd quartile, interquartile range, min., and max. in comparison to baseline (before oxygen exposure), which was set at 100%. (One-sample *t* test: ** *p* < 0.01, * *p* < 0.05, ns = non-significant).

Thus, we assessed the percent of MPs expressing proteins specific to platelets (CD41)—see Figure 2, neutrophils (CD66b)—see Figure 3, endothelial cells (CD146)—see Figure 4, and microglia (TMEM119)—see Figure 5.

The response pattern of MPs expressing platelet-specific CD41 after different oxidative stressors shows an ambivalent trend that approaches a sinusoidal pattern following PO_2_ increase. Every oxygen level increases CD41+ expression except 15% (ns) and an opposite reaction (a decrease) is elicited for 1.4-ATA exposure.

Neutrophil responses linked to inflammatory reactions show an interesting response pattern. Normobaric hyperoxia exposures share the same trend—mainly an increase of CD66b+. This is also the case for the 10% hypoxic stimulus, however with a smaller magnitude. Interestingly, the 1.4-ATA exposure again elicited an opposite reaction, suggesting a sort of “inhibitory” action.

CD146+ is a protein expressed by endothelial cells, which are known to react to oxidative stress but also to increased hydrostatic pressure [52]. It is interesting to note that hypoxic breathing gives a scattered response, although not reaching statistical significance—except for 10% and 1.4-ATA exposures, which elicited a significant reduction.

Microglia-derived responses (TMEM119+) show a global increase, except for 1.4 ATA; another example of varied reactions elicited by this specific PO_2_.

### 2.3. Microparticles Expressing Proteins from Cell Activation after One Hour of Different Oxygen Exposures

**Figure 6:**
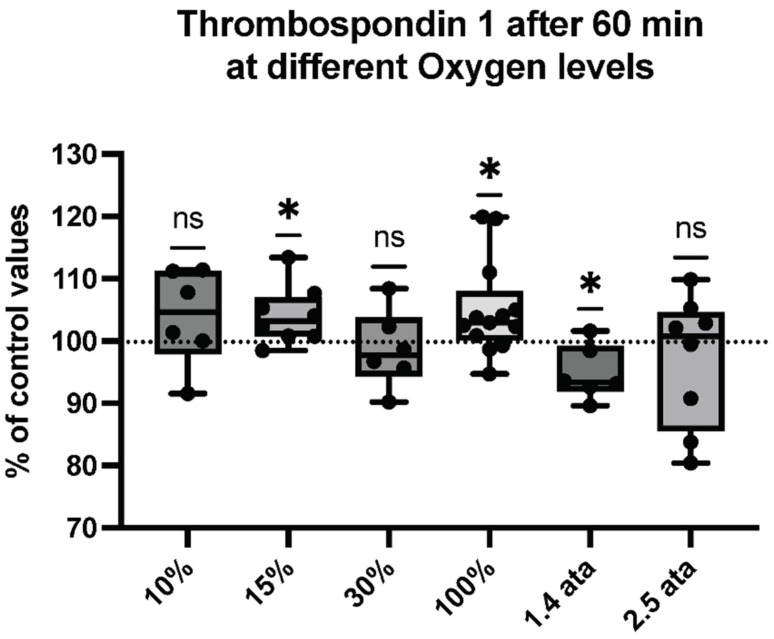
Thrombospondin-1 response following different oxygen levels exposure. Box and Whisker plots indicating median, 1st quartile, 3rd quartile, interquartile range, min., and max. in comparison to baseline (before oxygen exposure), which was set at 100%. (One-sample *t* test: * *p* < 0.05, ns = non-significant).

**Figure 7:**
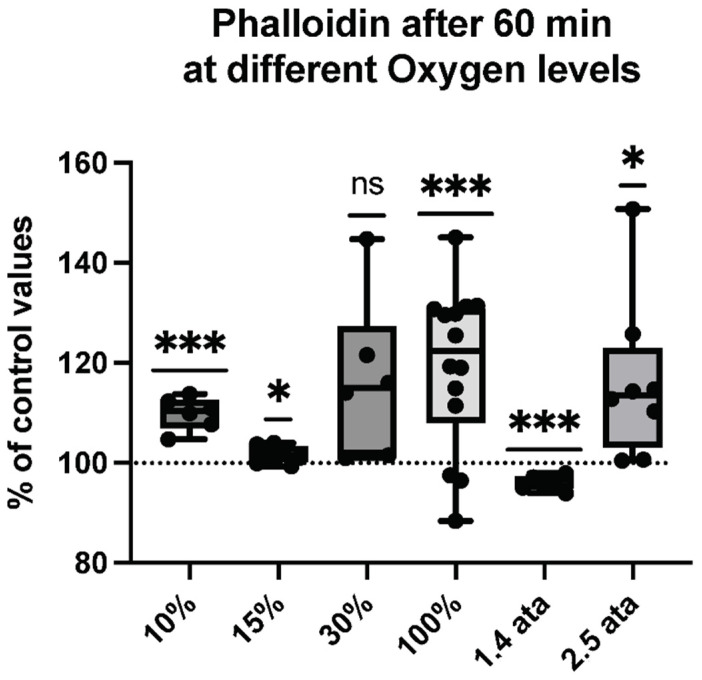
Phalloidin response following different oxygen levels exposure. Box and Whisker plots indicating median, 1st quartile, 3rd quartile, interquartile range, min., and max. in comparison to baseline (before oxygen exposure), which was set at 100%. (One-sample *t* test: *** *p* < 0.001, * *p* < 0.05, ns = non-significant).

MPs expressing thrombospondin-1 (TSP) (see Figure 6) and those binding phalloidin (See Figure 7) were also evaluated, seeking evidence for particles arising from cell activation. Phalloidin binding, a manifestation of membrane surface filamentous (F-) actin expression, occurs on particles released by activated neutrophils and possibly other cells, and TSP can be released by activated platelets and astrocytes [53,54,55].

Cellular reactions from platelets and astrocytes may demonstrate a neurovascular reaction of the body to cope with oxidative stress. Again, all oxygen levels react with an increment or without significant change, except for the 1.4 ATA, which shows a limited but significant decrease.

Cellular reactions eliciting filamentous actin liberation are extremely variable, but clearly demonstrate a membrane stress, and again the only reduction is found after 1.4-ATA exposure.

### 2.4. Percentual and Absolute Changes of Microparticles Expressing Proteins after One Hour of Different Oxygen Exposures

Significant elevations across many exposures were identified for CD41, CD66b, TMEM119, and phalloidin binding, whereas few changes were found for CD146 and TSP. 

**Figure 8:**
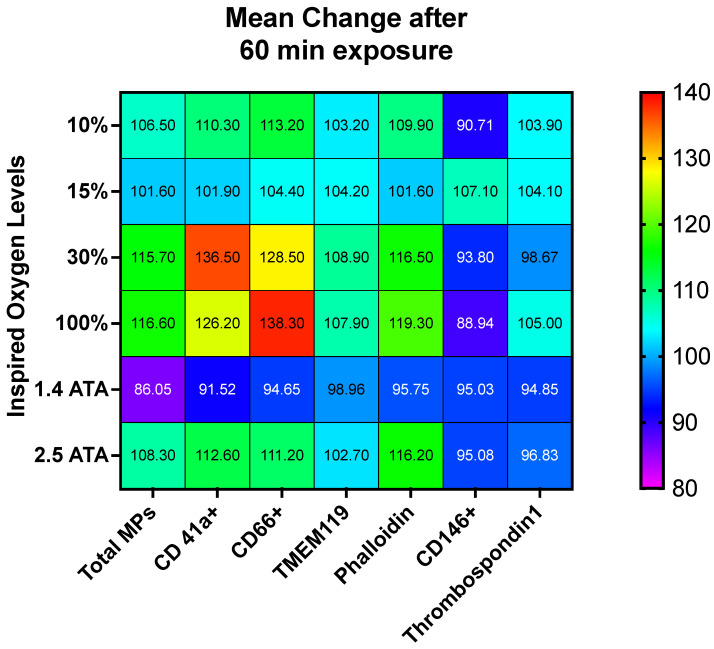
Percentual variations in MPs after 60 min of oxygen breathing. Levels of oxygen are shown on the ordinate, and Total MPs and MP sub-types are shown on the abscissa. Blood sampling occurred 120 min after exposures (in total 48 subjects participated to the experiment). Results are expressed in the heat map as mean percentage change.

The magnitude and direction of changes among all MP sub-types are illustrated as a heat map in Figure 8, while absolute values are presented in Table 1. It should be noted that when adding each % change in MPs, as shown in Table 1, the sum exceeds 100%. This is a common finding that is thought to indicate that MPs collide and share antigens [27,56]. 

## 3. Discussion

DCS is mostly known as an occupational risk for SCUBA divers or caisson workers (chamber hyperbaric technician, tunnellers), but also concerns space flight and extra-vehicular activity involving decompression from 1 ATA to 0.3 ATA in space-suit use [57], or altitude exposure up to 8000 m with an estimated probability of 0.2% [58] to 15–20% [59], respectively. Given its potential deleterious outcome, it must be prevented, especially in the occupational setting.

As mentioned earlier, OPB is one possible strategy. Unfortunately, available protocols are varied and complex, involving intervals of exercise while breathing O_2_ at partial pressures from 1 to 0.3 ATA for over more than 24 h [60]. The rationale behind the protocol is denitrogenating the astronaut to prevent the supersaturation of inert gas and subsequent DCS. 

However, this hypothesis does not provide a full explanation for phenomena like the variability between bubblers and non-bubblers, the bi-phasic mechanism of VGE expansion, increased VGE formation with depth, potential endothelial injury, or the presence of MPs [57,61]. 

While the role of MPs in decompression stress is not clear, they seem to play a major role as VGE precursors or as a mediator of inflammation [62]. To the best of our knowledge, this is the first study describing blood-borne MP responses to different PO_2_. Although net changes are a balance between production/liberation to the blood stream and sequestration/removal, we interpret differences in the patterns among the MP subsets elicited as reflecting the propensity for production because alternative cell types have different O_2_ tolerances and there is little evidence for the selective uptake of circulating MPs [32].

The first interesting results from our data are that the complex pattern of changes in MP numbers approximate a sinusoidal curve with nodes of nominal change in total number at 15% O_2_ and 1.4 ATA. This is consistent with the oxygen-sensing mechanism within the body. In case of hypoxia, hypoxia-inducible factors (HIFs) activate the transcription of numerous target genes that mediate both adaptive and maladaptive responses, including erythropoiesis, angiogenesis, metabolic reprogramming, or cardiovascular disease [1], while hyperoxia involves the production of ROS, which initiate signaling via the modulation of many molecules, such as NF-E2, Nrf2, or NF-kB [63]. At the same time, hyperoxia elicits an antioxidant scavenging adaptative response that can mimic the effect of hypoxia. Indeed, a sudden and sustained decrease in tissue oxygen tension, even in the absence of hypoxia (e.g., after hyperoxic oxygen breathing), acts as a trigger for HIF liberation and subsequent transcription [64,65].

Secondly, we anticipated that some changes in specific MP numbers are due to oxidative stress at the extremes of hypoxia and hyperoxia, as demonstrated by the elevations in plasma TSP levels [54]. However, the trade-off between both conditions is not obvious. Neutrophil MP generation was most prominent at 30% and 100%. Similarly, an intimate balance exists between the redox state and platelet activation, which may be reflected by MPs expressing CD41 [66]. On this particular point, previous work on hip replacement surgery, and randomized Oxygen administration one hour per day compared to one hour of air, showed a significant reduction of transfusions in the oxygen group, and also an increase of reticulocytes, both outcomes possibly resulting on the one hand due to CD41 increase and better bleeding reduction [67], and on the other hand, due to the normobaric oxygen paradox [6].

The same observation may apply to TMEM119, a fixed macrophage-like leukocyte resident within the central nervous system (CNS) [68]. All these features are consistent with a pro-inflammatory response related to oxygen breathing but does not concern the 1.4-ATA exposure that exhibits an inhibitory rather than an activation pattern. One possible explanation pertains to F-actin instability that occurs at high O_2_ partial pressures, thereby leading to the impairment of MP formation [69]. Alternatively, since phalloidin-binding MPs seem to play a role in tissue damage associated with DCS and can be generated by leukocytes [53,54], this can be interpreted as demonstrating membrane stress that is not found after 1.4-ATA exposure.

Despite the limitations to our study, including the observational, non-randomized trial design, hidden processes because of unmeasured confounders and the small sample sizes that may have altered the resulting patterns, these results question the unique profile of 1.4-ATA exposure. This is a reasonable interpretation since the end-points were objective and the participating research subjects served as their own control. Therefore, a clearer understanding of hyperoxia-induced signal transduction pathways is crucial to facilitate the design of successful therapeutic strategies as well as prevention strategies, such as OPB. 

Indeed, this investigation poses numerous questions as to the impact of O_2_ partial pressure on MPs. Regarding OPB, the production of MPs is maximal when breathing 100% oxygen, which could constitute a risk. However, it must be put into perspective. De-nitrogenation is a clear benefit while the kinetics of the production and elimination of MPs are unknown, with multiple O_2_ partial pressures being used during OPB. The results from this investigation also highlight questions such as the presumed innocuity of sham procedures in HBOT research [5].

Therefore, elucidating the mechanisms for changes and subsequent applications will require substantial future effort. Further experiments will also need to investigate the specific compensatory reactive adaptations at longer periods of pulsed hyperoxia.

## 4. Materials and Methods

### 4.1. Experimental Protocol

After written informed consent, 48 healthy non-smoking subjects (32 males and 16 females) volunteered for this study. None of them had a history of previous cardiac abnormalities or were under any cardio or vaso-active medication. The sample age was 43.7 ± 13.4 years old; height was 172.7 ± 10.07 cm; and weight was 68.4 ± 15.7 kg.

All experimental procedures were conducted in accordance with the Declaration of Helsinki [70] and approved by the Ethics Committee approval from the Bio-Ethical Committee for Research and Higher Education, Brussels (N° B200-2020-088). 

**Figure 9:**
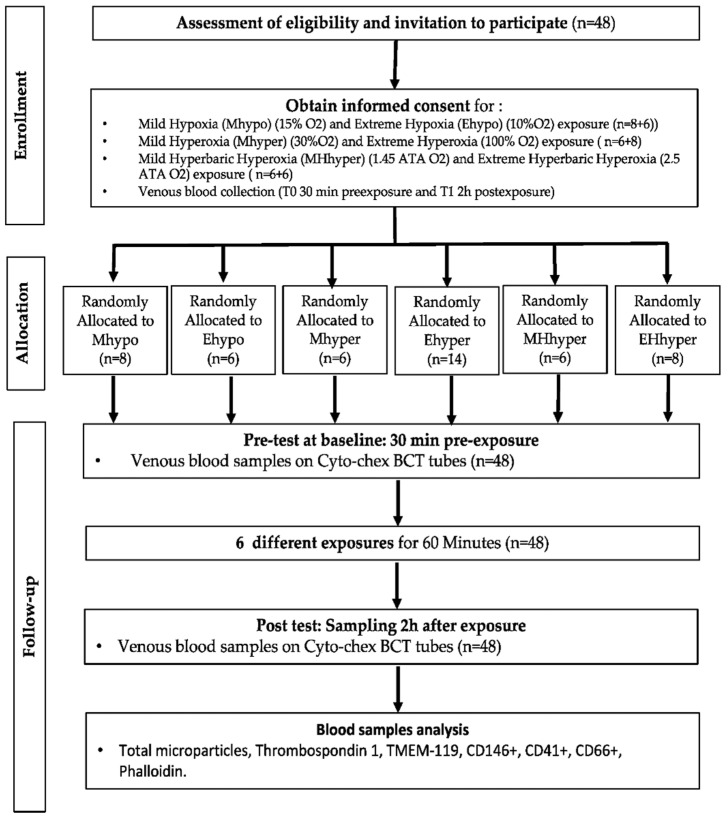
Experimental flowchart.

Participants were prospectively randomized into 6 groups of 6–14 persons each (Figure 9).

Subjects breathed different oxygen levels for a total duration of 60 min delivered by an oro-facial mask (non-rebreather mask) with a reservoir for the 10%, 15%, 30%, and 100% exposures, or a dedicated mask adapted to the hyperbaric environment (1.4 and 2.5 ATA) (Adult Silicone Mask–Laerdael, Laerdal Benelux, Vilvoorde, Belgium) were proposed.

The hypoxic gas supplies (10% and 15%) were achieved using a hypoxia generator and calibrated to reach the chosen level of oxygen (HYP 123, Hypoxico–Hypoxico Europe GmbH, Bickenbach, Germany). Normobaric hyperoxia was delivered using generated free-flow oxygen or medical oxygen tanks. Every exposure flow was calibrated by means of an oxymeter (Solo-O_2_ Divesoft, Halkova, Czech Republic) in the mask used by the subject to ascertain that the desired oxygen level was reached. Hyperbaric exposures were performed at the Hyperbaric Centre, Queen Astrid Military Hospital, 1120 Brussels, Belgium.

### 4.2. Blood Sampling and Laboratory Procedure

Blood samples were obtained before and 120 min after the different oxygen breathing sessions, the chosen time windows were achieved according to our previous work showing a clear variation in MP numbers 120-min post-exposure [71]. Blood (~5 mL) was drawn into Cyto-Chex BCT test tubes that contain a proprietary preservative (Streck, Inc., Omaha, NE, USA). Samples were sent by express mail to the University of Maryland (Dr. Thom) laboratory where all analyses were performed by following published techniques described in previous publications [54,72,73]. In brief, blood was centrifuged for 5 min at 1500× *g*, the supernatant was added to 12.5 mmol/L EDTA to impede MP aggregation, and then centrifuged at 15,000× *g* for 30 min. Aliquots of the 15,000× *g* supernatant were stained with antibodies for MP analysis by flow cytometry. Total MPs and sub-types were assayed in an 8-color, triple laser MACSQuant (Miltenyi Biotec Corp., Auburn, CA, USA) flow cytometer with the manufacturers’ acquisition software using standard methods, including a “fluorescence minus one control test” [73]. This analysis provides a way to define the boundary between positive and negative particles in an unbiased manner by defining the maximum fluorescence expected for a given subset after outlining the area in a two-dimensional scatter diagram when a fluorophore-tagged antibody is omitted from the stain set. This analysis allows a simple decision as to where to place the upper boundary for non-staining particles in a fluorescence channel. We define MPs as annexin V-positive particles with diameters from 0.3 to 1 µm. All supplies, reagents, and manufacturer sources have been described in previous publications [46,47].

### 4.3. Statistical Analysis

The normality of the data was verified by means of the Shapiro–Wilk test. Since a Gaussian distribution could be verified, crude data were analyzed by means of a paired *t*-test. When compared to air-breathing control values with the baseline measures set as 100%, changes were calculated for each exposure protocol and analyzed with a one-sample *t* test to allow an appreciation of the magnitude of change rather than the absolute values. All statistical tests were performed using a standard computer statistical package, GraphPad Prism version 9.00 for Mac (GraphPad Software, San Diego CA, USA). A threshold of *p* < 0.05 was considered statistically significant. All Table 1 data are presented as mean ± standard deviation (SD) and the figures are presented as box and whisker plots of median and quartiles.

## Figures and Tables

**Table 1:** Absolute values for microparticle-derived responses (MPs/μL). Results are given in mean ± SD. (Paired *t*-test: **** *p* < 0.0001, *** *p* < 0.001, ** *p* < 0.01, * *p* < 0.05).

Exposition	Baseline	After 120 min	*p* Value	*n*
Extreme Hypoxia 10% (0.1 ATA)	MPs/μL	Mps/μL	Paired *t* Test	
Total MPs	2241 ± 77.5	2388 ± 101.4	<0.0001 ****	6
Thrombospondin 1	12 ± 0.9338	12.44 ± 0.9422	0.2862	6
TMEM119	26.97 ± 0.4024	27.84 ± 0.8396	0.0480 *	6
CD 146+	33.11 ± 2.934	30.31 ± 2.329	0.0260 *	6
CD 41a+	4.5 ± 0.1117	4.962 ± 0.2962	0.0125 *	6
CD 66b+	13.56 ± 1.006	15.33 ± 0.9346	<0.0001 ****	6
Phalloidin	14.24 ± 0.6746	15.66 ± 0.9320	0.0007 ***	6
Moderate Hypoxia 15% (0.15 ATA)				
Total MPs	2085 ± 79.27	2114 ± 80.54	0.6174	8
Thrombospondin 1	18.28 ± 0.6066	19.03 ± 0.9983	0.0473 *	8
TMEM119	33.96 ± 0.4660	35.36 ± 1.141	0.0234 *	8
CD 146+	33.94 ± 0.5551	36.32 ±2.880	0.0156 *	8
CD 41a+	10.60 ± 1.136	10.64 ± 1.008	0.9582	8
CD 66b+	22.26 ± 0.5924	23.20 ± 1.216	0.1563	8
Phalloidin	20.60 ± 0.2195	20.93 ± 0.1674	0.0315 *	8
Moderate Hyperoxia 30% (0.3 ATA)				
Total MPs	1838 ± 123.2	2116 ± 68.66	0.0159 *	6
Thrombospondin 1	17.86 ± 1.4	17.55 ± 0.8321	0.0473 *	6
TMEM119	30.98 ± 2.081	33.60 ± 0.3714	0.0203 *	6
CD 146+	33.13 ± 2.933	30.94 ± 1.841	0.0472 *	6
CD 41a+	8.227 ± 0.7471	11.06 ± 1.192	0.0149 *	6
CD 66b+	17 ± 1.849	21.64 ± 0.5708	0.001 ***	6
Phalloidin	17.91 ± 2.438	20.54 ± 0.2030	0.0356 *	6
Hyperoxia 100% (1 ATA)				
Total MPs	1786 ± 118.0	2072 ± 56.29	<0.0001 ****	14
Thrombospondin 1	17.16 ± 0.5448	18.01 ± 1.218	0.0266 *	14
TMEM119	30.80 ± 1.993	33.10 ± 0.5293	0.0031 **	14
CD 146+	42.38 ± 12.31	35.32 ± 4.333	0.0785	14
CD 41a+	7.751 ± 0.9495	9.552 ± 1.086	0.0035 **	14
CD 66b+	14.85 ± 1.662	20.30 ± 1.723	<0.0001 ****	14
Phalloidin	16.74 ± 2.008	19.65 ± 0.7498	0.0023 **	14
Hyperbaric Hyperoxia 1.4 ATA				
Total MPs	2766.69 ± 80.74	2381.7 ± 156.3	0.0004 ***	6
Thrombospondin 1	11.82 ± 0.26	11.21 ± 0.4	0.0354 *	6
TMEM119	31.27 ± 0.35	30.94 ± 0.36	0.087	6
CD 146+	22.62 ± 0.3	21.5 ± 0.32	0.0011 **	6
CD 41a+	6.56 ± 0.40	5.6 ± 0.22	0.0336 **	6
CD 66b+	17.65 ± 0.18	16.7 ± 0.4	0.0077 **	6
Phalloidin	16.86 +/− 0.22	16.15 +/− 0.09	0.001 ***	6
Hyperbaric Hyperoxia 2.5 ATA				
Total MPs	1846 ± 128.8	1989 ± 44.05	0.0206 *	8
Thrombospondin 1	17.08 ± 2.048	16.37 ± 0.865	0.6406	8
TMEM119	32.13 ± 0.445	32.98 ± 0.5293	0.0347 *	8
CD 146+	39.47 ± 4.985	36.88 ± 6.047	0.3828	8
CD 41a+	8.114 ± 0.6390	9.109 ± 0.5885	0.0078 **	8
CD 66b+	17.64 ± 0.8149	19.59 ± 1.327	0.0057 **	8
Phalloidin	16.80 ± 1.893	19.22 ± 0.4839	0.0149 *	8

## Data Availability

Data are available at request from the authors.

## Abbreviations

MPs	Blood Borne Microparticles
OPB	Oxygen Pre-Breathing
VGE	Vascular Gas Emboli
DCS	Decompression Sickness
NF-kB	Nuclear Factor kappa-light-chain-enhancer of activated B cells
NRF2	Nuclear Factor Erythroid 2 Related–Factor 2
PO_2_	Oxygen Partial Pressure
TSP	Thrombospondin 1
NF-E2	Nuclear Factor, Erythroid 2
HIFs	Hypoxia Inducible Factors
